# Efficiency Assessment of Breast Clinics for Patients Under 35: A Comparative Analysis of Targeted Models in a University Hospital

**DOI:** 10.7759/cureus.54428

**Published:** 2024-02-18

**Authors:** Ona O Fagbemi, Charles Ojo, Maryam A Khan, Sekhar Marla, Sankaran Narayanan, Sadaf Jafferbhoy, Soni Soumian

**Affiliations:** 1 Department of General Surgery, University Hospitals of North Midlands NHS Trust, Stoke-on-Trent, GBR; 2 Department of Emergency Medicine, United Lincolnshire Hospitals NHS Trust, Boston, GBR; 3 Department of Breast Surgery, East Cheshire NHS Trust, Macclesfield, GBR; 4 Department of Breast Surgery, University Hospitals of North Midlands NHS Trust, Stoke-on-Trent, GBR

**Keywords:** benign breast diseases, breast surgery, ultrasound, under 35 years of age, breast disease

## Abstract

Despite the higher incidence of breast cancer in older age groups, it remains pertinent not to overlook breast cancer occurrence in those aged 35 years and below. Recent transitions toward targeted under-35 clinics in England aim to enhance efficiency and meet referral standards. Three models were planned, and we assessed the efficiency of each model. This study, conducted for five months within a single National Health Service (NHS) trust, analyzed data from the following clinics: the General One-Stop Clinic, the Under 35 One-Stop Clinic with ultrasound services (USS), and the Under 35 Clinic without USS services. Of the 300 patients recruited (100 consecutive patients from each clinic), 94.3% were female. The average age at presentation was 27.53 years. The most frequently encountered age group was between 26 and 30 years, and the majority of patients had palpable lumps (78, 51.6%). Out of 300 patients who attended the clinics, 151 had USS, and of these, 15 biopsies were performed. Fibroadenomas (32, 21.2%) and cysts (22, 14.6%) were the most common radiological findings. We found that more breast imaging was being undertaken for under-35 patients who attended the general one-stop clinics compared to the specific under-35 clinics. Targeted breast clinics for individuals 35 years and below offer an effective approach in terms of resource allocation and meeting cancer targets.

## Introduction

Breast health is a critical aspect of women’s overall well-being, and early detection of breast cancer significantly improves prognosis and survival rates [1]. Although it is established that older age groups are at the highest risk for breast cancer [2,3], it is important to not overlook the incidence in women aged 35 and below.

In recent years, several hospitals in England have transitioned toward targeted under-35 clinics for breast care and were recommended in the guides from the National Health Service (NHS) National Cancer Action Team to increase efficiency, reduce backlogs, and ensure implementation of the symptomatic 2ww referrals standard of 2010 [4]. At our trust, we trialed three different pathways involving three clinics for under 35 patients. In addition to the general one-stop clinic, catering to patients of all ages referred under the two-week pathway, we had two dedicated under-35 clinics run by a clinician, one with ultrasound services (USS) and a parallel clinic that did not offer same-day USS services. 

At present, there is limited data regarding the specific outcomes of these targeted breast clinics for people under 35 years old in the United Kingdom. In this study, we aimed to assess the outcome of these clinics. 

## Materials and methods

In this retrospective study, we systematically reviewed patient records spanning five months from December 2021 to May 2022 within a single National Health Service (NHS) trust. The investigation was centered on patients aged 35 years and younger, encompassing three specialized clinics, including the general one-stop clinic (consisting of all patients attending the two-week wait clinics regardless of age), the under-35 one-stop clinic with USS services, and the under-35 clinic without USS services. 

Following a uniform and methodical approach, the recruitment plan entailed enrolling 100 patients from each clinic in succession. All patients who fell within this number were included. The data collection process employed a predefined uniform structured data collection template, which was collectively agreed upon by the team. We recorded the following key characteristics, demographics, and clinical features (symptoms and signs) at the primary care level and with the breast clinicians, USS findings, biopsy outcomes, and long-term follow-up data. Subsequently, following data collection, the analytical phase was executed using Microsoft Excel. 

We ensured strict adherence to patient confidentiality and data anonymization.

## Results

A total of 300 patients met the inclusion criteria for the study, with 100 patients from each of the aforementioned clinics, and of this number, 283 (94.3%) were females. The average age at presentation was 27.53 years, and the median age was 28 years. The number of individuals under 25 was lower compared to the other age groups (Table 1).

**Table 1:** Age and sex at the initial clinic visit.

Age (years)	Sex		Total, *n* (%)
	Female, *n* (%)	Male, *n* (%)	
<25	81 (91.0%)	8 (9.0%)	89 (58.9%)
26-30	104 (97.2%)	3 (2.8%)	107 (35.7%)
31-35	98 (94.2%)	6 (5.8%)	104 (34.7%)
Total	283 (94.3%)	17 (5.7%)	300 (100.0%)

**Figure 1:**
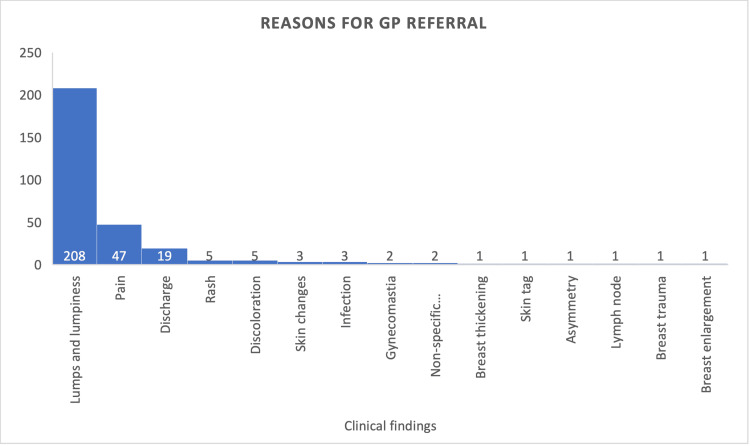
Reasons for referral from primary care. Illustrates the reasons for referral of all 300 patients from primary care centers to breast clinicians.

The reasons for referral from primary care were mostly due to discrete lumps, which were followed by pain and then nipple discharge (Figure 1).

Approximately half of the referred patients (151) underwent USS, with the age group 31-35 having the highest percentage of requests (62, 41.1%) (Table 2). Among the remaining patients without USS (149), 146 were discharged on the same day, while three had follow-up appointments.

**Table 2:** USS request rate total. USS, ultrasound service

Age range (years)	*n *(%)
<25	35 (23.1%)
26-30	54 (35.7%)
31-35	62 (41.1%)
Total	151

Also regarding this group who did not have USS, 83 of them were originally referred from the primary care center due to lumps and lumpiness, and of this number, 76 (88.3%) had no significant clinical findings that needed further evaluation.

**Figure 2:**
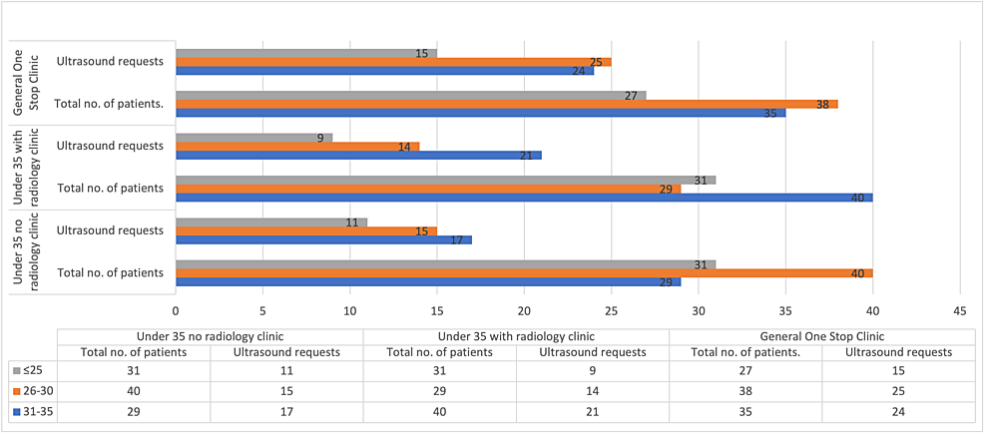
USS request rates across clinics by age group. The figure details the number of visits to each clinic by age group, which totals 100, respectively. It further shows the rate of USS requests from each clinic. USS, ultrasound service

Looking at the distribution among clinics, the general one-stop clinic had the highest USS request rate (64%). The number of USSs done in the other two clinics was 43% and 44%, respectively (Figure 2).

The indications for the USS by the breast clinicians varied from lumps to lumpiness, nipple discharge, palpable nodes, etc. (Table 3).

**Table 3:** Clinical findings/indications for USS. ^*^Others included patient request, discharge, eczema, lactational changes, fuller breast, ptotic breast, areola indentation, nipple induration, muscular thickening, and nonspecification. USS, ultrasound service

Clinical findings	Number of patients, *n* (%)
Discrete lump (with/without pain)	78 (51.6%)
Lumpiness	47 (31.1%)
Nipple discharge	9 (6.0%)
Palpable nodes	5 (3.3%)
Accessory breast tissue	2 (1.3%)
Others*	10 (6.6%)
Total	151

In terms of radiological diagnosis, fibroadenomas were the most frequently detected, with 32 out of 151 patients who underwent USS (21.2%). This was followed by cysts in 22 patients (14.6%) and infections/collections in five patients (3.3%) (Table 4). In cases of clinicians’ suspicion of lumpiness (47 cases), three patients had cysts and two were diagnosed with fibroadenoma radiologically. Among those who had discrete lumps (78, 51.6%), 30 of these lumps were fibroadenomas and 16 were cysts. Thus, out of the diagnosed lumps in the clinic, 59% (46 out of 78 patients) exhibited radiological findings consistent with either fibroadenomas or cysts. 

**Table 4:** Radiological diagnosis by age group.

Radiological diagnosis	≤25 years, *n* (%)	26-30 years, *n* (%)	31-35 years, *n* (%)	Total across clinics, *n* (%)
No anomalies	19 (23.5%)	28 (34.6%)	34 (41.9%)	81 (53.6%)
Fibroadenoma	8 (25%)	14 (43.8%)	10 (31.3%)	32 (21.2%)
Cysts	4 (18.2%)	8 (36.4%)	10 (45.4%)	22 (14.6%)
Infection/collection	1 (20%)	-	4 (80%)	5 (3.3%)
Galactocele	-	1 (100%)	-	1 (0.7%)
Unspecified lump	1 (33.3%)	1 (33.3%)	1 (33.3%)	3 (2%)
Lipoma	1 (33.3%)	1 (33.3%)	1 (33.3%)	3 (2%)
Sclerosing adenosis	-	1 (100%)	-	1 (0.7%)
Papilloma	1 (100%)	-	-	1 (0.7%)
Lactational hamartoma	-	-	2 (100%)	2 (1.3%)
Total	35	54	62	151

Following radiological diagnosis, only 15 patients had a biopsy, 9 (60%) of which were confirmed to be a fibroadenoma, one was an intraductal papilloma, and one cyst, with the rest being unspecified benign changes. In terms of interventions following USS, two patients had excision of fibroadenoma and the patient with intraductal papilloma had vacuum-assisted excision. Regarding follow-up, 95% of the patients were discharged from the pathway during the initial visit. The remaining patients included those offered interventions, individuals with eczema who had follow-up after corticosteroid use, and those treated for infections.

## Discussion

For this study, 300 patients were examined consecutively, who were referred from primary care. Most of the patients were referred because of lumps in association with or without other symptoms (208, 69.3%), followed by breast pain (47, 15.6%), and then nipple discharge (19, 6.3%) (Figure 1). The findings of lumps being the highest reason for referral are similar to other studies conducted [5-7]. Out of the lumps diagnosed in the breast clinic, 59% of those who had discrete lumps had radiological findings of either fibroadenomas or cysts. 

Regarding radiological outcomes, it was interesting to note that despite nodularity/lumpiness being the second highest reason for requesting USS (47 cases), only 10.6% of them had clinically significant findings on USS: three cysts and two fibroadenomas. The rest are findings of dense breast tissue or no anomaly. Previous studies have suggested that in women under 35 years if there is vague nodularity or asymmetric nodularity, the next step will be to repeat the clinical examination, possibly midcycle after one to two further menstrual cycles [8]. Therefore, it might be useful for primary care physicians to consider reviewing patients in the clinic for a repeat examination. The latest data available from the National Institute for Health and Care Research (NIHR) shows that the cost of a USS is about £101 [9]. It will thus enable better utilization of limited resources to have USS done in the under-35 age group with stricter criteria, going by the aforementioned findings.

A significant observation lies in the variance of USS request rates among the clinics. The general one-stop clinic exhibited the highest USS request rate at 64%, and the reason for this is not clear. It might be due to the higher numbers of clinicians, a larger volume of patients in the clinic, and increased availability of imaging modalities. Further corroborating this is that despite the absence of USS in the targeted under-35 clinics, compared to the 35 one-stop clinics with USS, the request rate remained comparable at 43% and 44%, respectively. There is no report showing this comparison in the literature.

Analysis of the clinical findings necessitating USS, as picked up by the breast clinicians, revealed a preponderance of discrete palpable lumps constituting 51.6% of presentations, aligning with established patterns of breast disease in which lumps are among the common signs at presentation (Table 3) [10-12]. In contrast, nipple discharge, palpable nodes, and accessory breast tissue were less frequent reasons.

The most common radiological finding in those with discrete lumps (78 patients) who had USS was fibroadenoma (30, 38.5%) as is expected with the younger population [13,14], followed closely by cysts (16, 20.5%). There were no malignancies identified in this cohort of patients. A study spanning one year analyzing the targeted under-35 clinic (1144 patients) showed an incidence of less than 1% [15]. It is important to state that a limitation of our study was the small sample size.

Among the subset of patients who underwent biopsy, fibroadenoma was the most predominant histological finding, accounting for 60% of those biopsied. If a patient under 35 is detected to have discrete lumps clinically, the chance of fibroadenoma according to our study is 38.4%, and if lumpiness is detected clinically, the chance of it being fibroadenoma is 4.3%.

Interventions were generally limited, with excision or aspiration procedures performed specifically for specific cases, aligning with local practice guidelines. Most of the patients in this cohort (95%) were discharged at the initial visit, and the remaining number of patients had follow-up in three months, to ensure complete treatment and/or relief of symptoms. Our clinical practices adhere to the recommendations of Getting It Right the First Time (GIRFT), particularly in the context of minimizing unwarranted outpatient visits and unnecessary surgical interventions [16].

This study presents valuable insights into the increased efficiency of the under 35 standalone clinics, but it is essential to acknowledge the limitations of this study. First, it is a retrospective study with a small number of patients and spanning a short duration of time. Thus, the findings may not be representative of the entire population. Also, being a single-center study, the recommendations we have put forward for running stand-alone clinics for those under 35 may not apply to all institutions. Larger prospective studies are needed to validate these findings further. 

## Conclusions

This study establishes the comparatively increased efficiency of the under-35 clinics when compared to the general one-stop clinic. The findings advocate for the establishment of a standalone model for under 35 clinics. Whether incorporating ultrasound scans or not, this model emerges as a potential solution for optimizing resource utilization in breast care in the under-35 age group.
